# A Self-Assessment Web-Based App to Assess Trends of the COVID-19 Pandemic in France: Observational Study

**DOI:** 10.2196/26182

**Published:** 2021-03-12

**Authors:** Fabrice Denis, Arnaud Fontanet, Yann-Mael Le Douarin, Florian Le Goff, Stephan Jeanneau, François-Xavier Lescure

**Affiliations:** 1 Institut Inter-régional de Cancérologie Jean Bernard Le Mans France; 2 Emerging Diseases Epidemiology Unit Institut Pasteur Paris France; 3 French Society of Digital Health Paris France; 4 Kelindi Lille France; 5 Adobis Group Grenoble France; 6 AP-HP, Infectious and Tropical Diseases Department Bichat-Claude Bernard University Hospital Paris France

**Keywords:** app, big data, COVID-19, diagnosis, diagnostic test, digital health, France, mobile phone, observational, participatory app, self-assessment, surveillance, trend, web-based app

## Abstract

**Background:**

We developed a self-assessment and participatory web-based triage app to assess the trends of the COVID-19 pandemic in France in March 2020.

**Objective:**

We compared daily large-scale RT–PCR test results to monitor recent reports of anosmia through a web-based app to assess the dynamics of emergency department visits, hospitalizations, and intensive care unit (ICU) admissions among individuals with COVID-19 in France.

**Methods:**

Between March 21 and November 18, 2020, users of the maladiecoronavirus.fr self-triage app were asked questions about COVID-19 symptoms. Data on daily hospitalizations, large-scale positive results on RT–PCR tests, emergency department visits, and ICU admission of individuals with COVID-19 were compared to data on daily reports of anosmia on the app.

**Results:**

As of November 18, 2020, recent anosmia was reported 575,214 times from among approximately 13,000,000 responses. Daily anosmia reports during peak engagement with the app on September 16, 2020, were spatially correlated with the peak in daily COVID-19–related hospitalizations in November 2020 (Spearman rank correlation coefficient [ρ]=0.77; *P*<.001). This peak in daily anosmia reports was observed primarily among young adults (age range 18-40 years), being observed 49 days before the peak of hospitalizations that corresponded to the first wave of infections among the young population, followed by a peak in hospitalizations among older individuals (aged ≥50 years) in November 2020. The reduction in the daily reports of anosmia associated with the peaks in the number of cases preceded the reduction in daily hospitalizations by 10 and 9 days during the first and the second waves of infection, respectively, although the reduction in the positivity rates on RT–PCR tests preceded the reduction in daily hospitalizations by only 2 days during the second wave of infections.

**Conclusions:**

Data on daily reports of anosmia collected through a nationwide, web-based self-assessment app can be a relevant tool to anticipate surges in outbreaks, hospitalizations, and ICU admission during the COVID-19 pandemic.

**Trial Registration:**

ClinicalTrials.gov NCT04331171; https://clinicaltrials.gov/ct2/show/NCT04331171

## Introduction

Apps involving patient-reported outcomes have been shown to improve outcomes including survival benefit [1-3]. We developed and launched a self-assessment and participatory web-based surveillance app for COVID-19, called “maladiecoronavirus.fr,” during the growth phase of the COVID-19 pandemic in March 2020 in France. This self-triage tool was aimed at directing symptomatic patients with COVID-19 to emergency care or to general practitioners after the analysis of symptoms and comorbidities. We previously reported that data from this web-based app could be a relevant tool to reduce the burden on emergency call centers [4]. Interestingly, this web-based app was useful in monitoring COVID-19 spread during the initial wave of infections in March 2020 in France, with spatial correlations among the number of hospitalizations, users reporting fever and cough, and users reporting anosmia [5]. The ability to detect an early rise and decline in COVID-19 incidence would also be extremely useful in anticipating a patient’s course of hospitalization to avoid overcrowding at emergency departments and saturation of the intensive care unit (ICU) and to anticipate the availability of hospital beds dedicated to patients with COVID-19.

In France, nationwide RT–PCR test results are available on a daily basis and are used to estimate the effective reproduction number. However, variability in testing indications and access to tests have led to speculations regarding the practicality and validity of large-scale RT–PCR test results and concomitantly the monitoring of trends of the pandemic. Our web-based app may be a useful alternative. In this study, we compared the results of large-scale RT–PCR tests on a daily basis to daily reports of anosmia from among the users of the app to predict the dynamics of emergency department visits, hospitalizations, and ICU admission of individuals testing positive for COVID-19 during the outbreak in France.

## Methods

Users of maladiecoronavirus.fr in France were recruited through nationwide media campaigns, including social media, radio, and magazine campaigns, during March 17-29, 2020. Participants were recruited through the maladiecoronavirus.fr website as previously described [5]. Data including sociodemographic characteristics, zip code, and comorbidities were anonymously obtained. The participants were asked about the following symptoms potentially associated with COVID-19: fever (body temperature of >37.7°C), unusual cough, shortness of breath, sore throat, muscle aches, diarrhea, anorexia, and asthenia. Anosmia was included in this list of potential symptoms on March 21, 2020. After recording the symptoms of the participants, a notification was sent to them recommending them to either stay at home and use the website again in case of evolving symptomatology (self-monitoring) or to contact a general practitioner or an emergency number if they reported experiencing dyspnea or severe anorexia. Questionnaires responses were excluded from the analysis if they did not include a zip code or if the duration of completion was inconsistent (<30 seconds). The study was approved by the French Health Data Hub, which reviews the ethical conduct of research with human subjects and the confidentiality and safety of their data. The web-based app was not considered a medical device by regulatory authorities because no tracking was performed, and the data were anonymized. The app did not monitor the adherence of the participants to the self-triage recommendations and did not inquire participants about their test results. Participants did not need to create an account or log into the app to access it. The app did not identify duplicate responses and did not make follow-up inquiries with the participants.

Data on daily large-scale positive outcomes on diagnostic RT–PCR tests, emergency department visits, hospitalizations, and ICU admissions among individuals with COVID-19 were obtained from Santé publique France, the Oscour network, and the French Ministry of Health. Big data were analyzed by Data Chain (Adobis Group). We did not ask app users for daily reports, but we assessed daily overall reports of anosmia among the users. We compared these daily reports of anosmia on the app, daily positive RT–PCR tests results, daily emergency department visits, daily conventional hospitalization, and daily ICU admissions among individuals with COVID-19 in order to determine which source of data best predicts the peaks and declines in hospitalization and ICU admission.

Age stratification was performed to assess the predictability of subsequent hospitalization. Spearman rank correlation analysis was performed for statistical analysis.

## Results

Between March 17 and November 18, 2020, a total of 13,000,343 completed questionnaires were included, of which 7,507,332 were excluded owing to the unavailability of the zip code or an inconsistent completion duration, and recent anosmia was reported 575,214 times. The number of assessed questionnaires represents the number of assessments and not the number of individuals.

**Figure 1:**
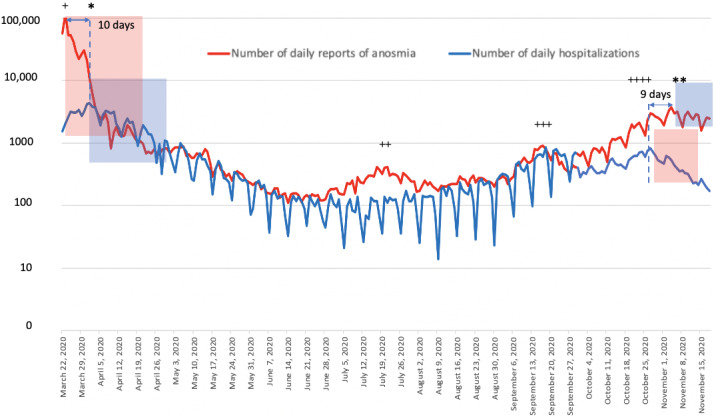
Comparisons between the daily number of hospitalizations and the daily reports of anosmia on the web-based self-triage app maladiecoronavirus.fr during the first and second peaks of the pandemic in France between March 21, 2020, and November 18, 2020. The first peak of hospitalizations occurred on January 4, 2020, and the second one occurred on April 11, 2020. In total, 4 peaks of daily reports of anosmia were recorded from the web-based app: March 23, 2020 (+), July 22, 2020 (++), September 16, 2020 (+++), and October 26, 2020 (++++). *Peak of hospitalizations during the first wave of the COVID-19 pandemic. **Peak of hospitalizations during the second wave of the COVID-19 pandemic. The graph shows a reduction in the number of connections 10 days before the reduction in daily hospitalizations during the first wave of the pandemic and 9 days before the reduction in daily hospitalizations during the second wave (semilog scale). Red transparent windows show the reduction in daily reports of anosmia, and blue transparent windows show the reduction in the number of daily hospitalizations during the first and second waves of the pandemic.

Data on daily reports of anosmia on the website and daily hospitalizations during the outbreak were well correlated (ρ=0.75; *P*<.001) (Figure 1). During the first wave of infections in March 2020, the peak in daily reports of anosmia (113,234 connections) was reached on March 22, 2020, and that of daily hospitalizations (4281 patients) was reached 10 days later on April 1, 2020.

**Figure 2:**
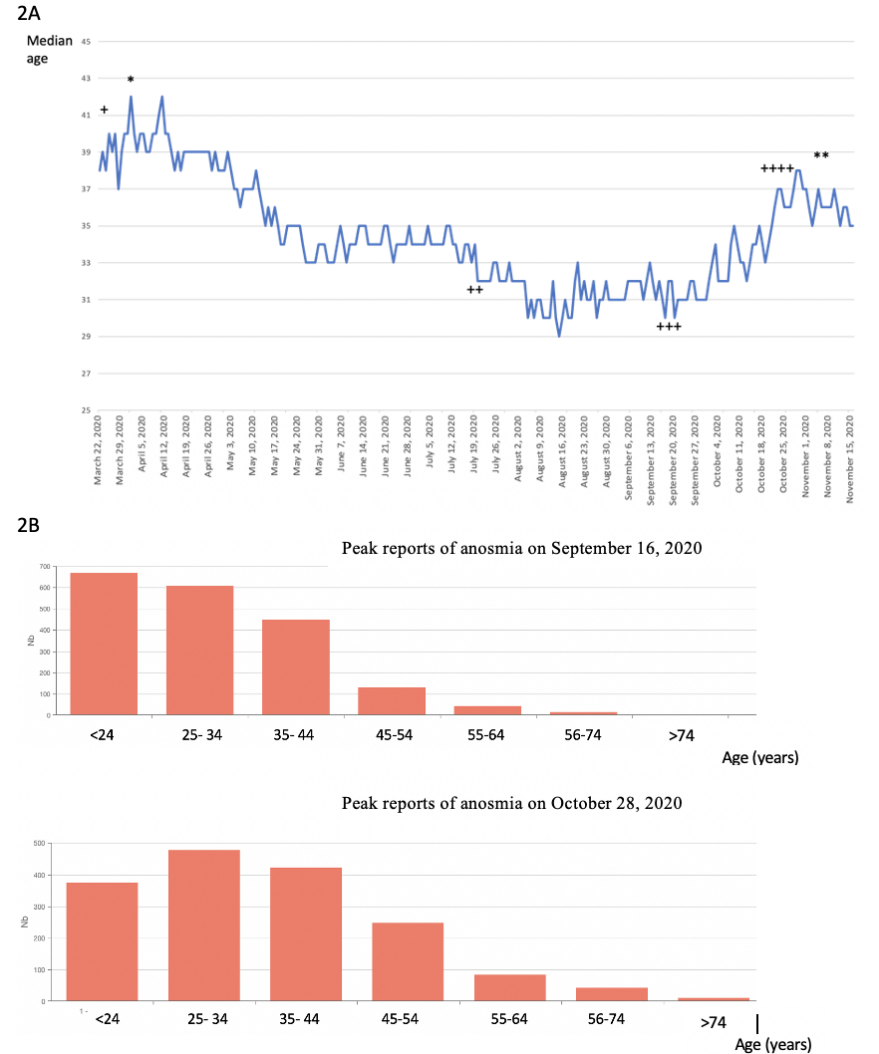
Age of users reporting anosmia on the web-based self-triage app maladiecoronavirus.fr during the COVID-19 outbreak in France. (2A) Median age of the users of the app. *Peak of hospitalizations during the first wave of the pandemic. **Peak of hospitalizations during the second wave of the pandemic. Four peaks of daily reports of anosmia were recorded from the web-based app: the first peak of reports of anosmia (+; median age=40 years), second peak of reports of anosmia (++; median age=32 years), third peak of reports of anosmia (+++; median age=30 years), and fourth peak of reports of anosmia (++++; median age=37 years). (2B) Histograms of the number of reports of anosmia based on the age of the users during third and fourth peaks of connections. Users of aged >45 years were more numerous in the October 2020 outbreak than in the September 2020 outbreak peak of during the second wave of hospitalizations (n=190/2088, 9.1% vs n=402/1668, 24.1% respectively).

Before and during the second wave of infections towards the end of October 2020, a total of 3 early peaks of daily reports of anosmia on the website occurred on July 21, 2020 (n=417); September 16, 2020 (n=905); and October 26, 2020 (n=805); and the peak in daily hospitalizations was reached on November 4, 2020 (n=3681), having occurred 49 days after the highest peak in the daily reports of anosmia. Daily hospitalizations decreased after November 4, 2020; this occurred 9 days after the last peak in daily reports of anosmia. Data on the median age of users of the web-based app are shown in Figure 2. The median age of the participants was 40 years (IQR 27-56) during the first peak of connections (first wave of the pandemic in March 2020); 32 years during the second peak of connections on July 22, 2020; 30 years during the third peak of connections on September 16, 2020; and 37 years during the fourth peak of connections on October 26, 2020 (second wave of the pandemic). Participants aged ≥45 years were more numerous during the last peak of connections in October 2020 than in third peak in September 2020 (n=190/2088, 9.1% vs n=402/1668, 24.1%, respectively) (Figures 2A and 2B).

**Figure 3:**
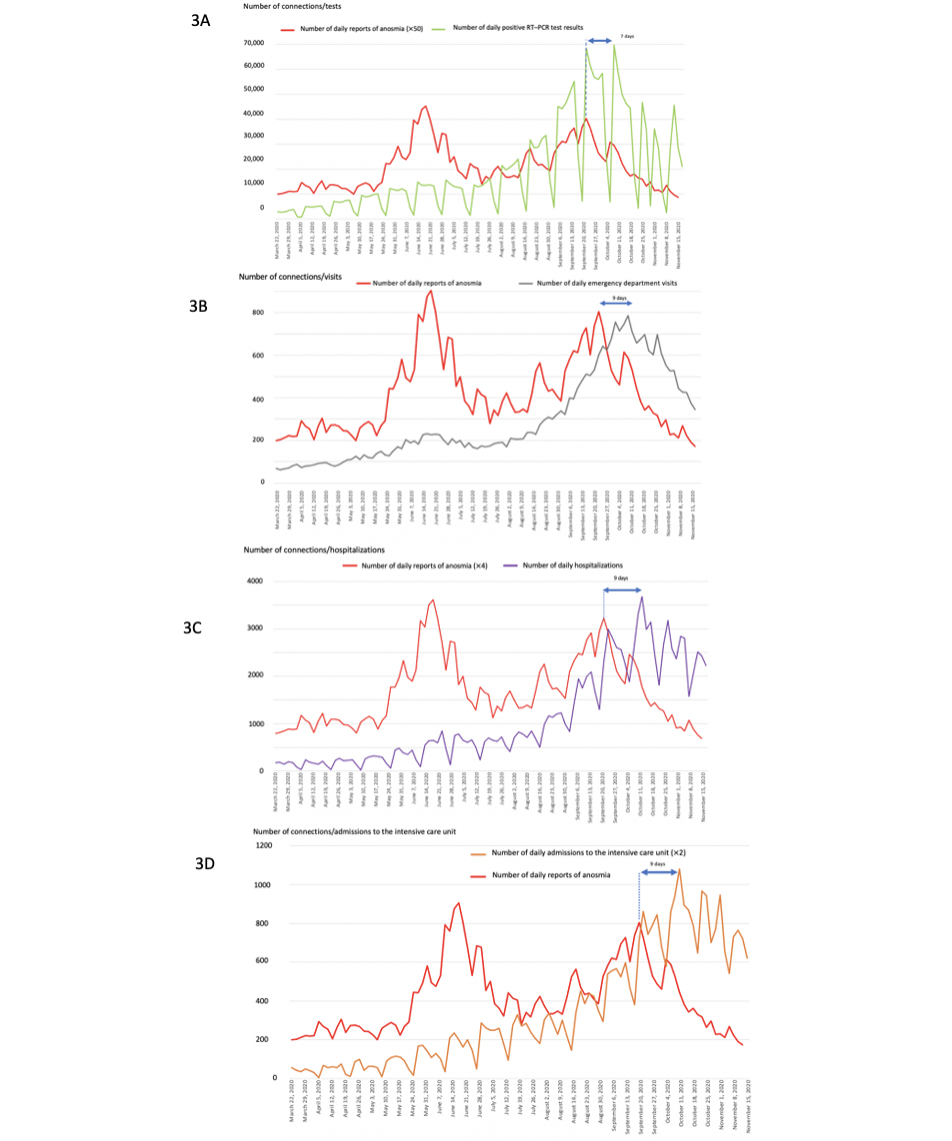
Trends of the COVID-19 pandemic during the second wave of infections in France from August 10 to November 18, 2020: (3A) comparison between the number of daily reports of anosmia and positive outcomes on daily RT–PCR tests; (3B) comparison between the number of daily reports of anosmia and the number of emergency department visits; (3C) comparison between the number of daily reports of anosmia and the number of daily hospitalizations; and (3D) comparison between the number of daily reports of anosmia and the number of daily admissions to the intensive care unit.

The number of ICU admissions peaked (n=450) on November 4, 2020, 9 days after the last peak of daily reports of anosmia. Daily numbers of emergency department visits peaked (n=787) on November 2, 2020, a total of 7 days after the last peak of daily reports of anosmia. The number of positive outcomes on daily RT–PCR tests peaked (n=69,564) on November 2, 2020, a total of 7 days after the last peak of daily reports of anosmia and only 2 days before the peak of hospitalization occurred (Figures 3A, 3B, 3C, and 3D).

A large-scale curfew, followed by a general lockdown, was initiated on October 23, 2020. Contamination in RT–PCR tests reduced on November 2, 2020, a total of 15 days after lockdown enforcement, and daily reports of anosmia decreased from October 27, 2020, a total of 4 days after lockdown enforcement.

**Figure 4:**
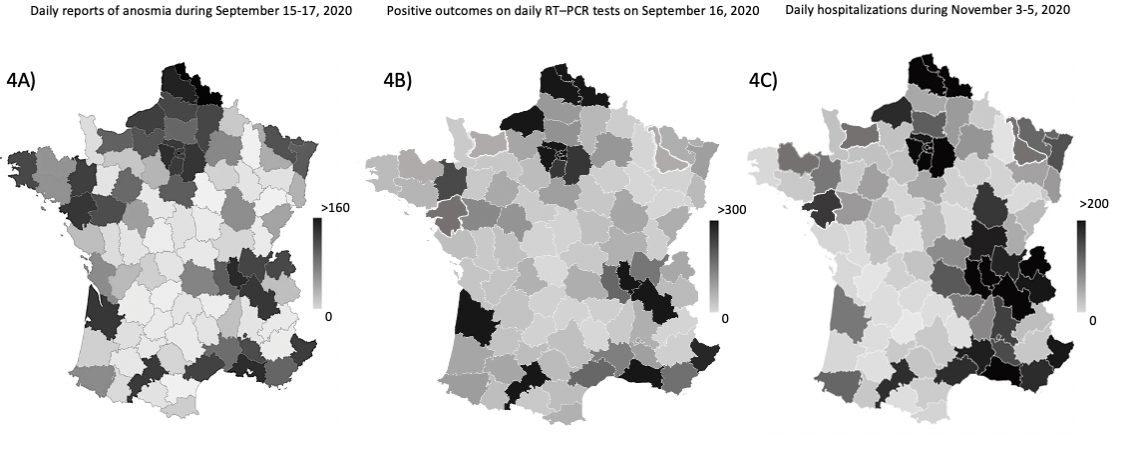
Maps displaying the correlation between daily reports of anosmia with COVID-19–related hospitalizations during the second wave of the pandemic in France. The cumulative number of (4A) daily reports of anosmia during September 15-17, 2020; (4B) positive outcomes on large-scale RT–PCR tests on September 16, 2020; and (4C) daily hospitalizations of individuals with COVID-19 in France during November 3-5, 2020.

Large-scale positive outcomes on daily RT–PCR tests and daily reports of anosmia during the third peak of connections on September 16, 2020, were spatially correlated at the county level with the peak in daily COVID-19–related hospitalizations in November 2020 (ρ=0.77 for both; *P*<.001) (Figure 4).

## Discussion

Our results suggest that the peak of daily reports of anosmia determined from our web-based self-triage app based on symptoms reported by individuals suspected with COVID-19 in September 2020 was spatiotemporally correlated with the daily peak of hospitalizations during the second wave of the pandemic in November 2020 in France. During this period, an early first peak of daily reports of anosmia occurred among young adults on July 21, 2020, and the highest peak occurred on September 16, 2020, occurring 49 days before the peak of daily hospitalizations in November 2020. A reduction in the number of daily reports of anosmia by users of the web-based app also preceded the reduction in the number of daily hospitalizations by 10 and 9 days during the first and the second waves of the pandemic, respectively, in France.

During the first peak of hospitalizations in March 2020, we initially observed that the peak of daily reports of anosmia on our web-based app occurred 10 days before the peak in daily hospitalizations, which is similar to the mean 11-day period between infection and hospitalization among individuals experiencing severe forms of the disease as reported previously [6]. We did not assess the dynamics of RT–PCR tests in the population in March 2020 because RT–PCR tests were not performed on a large scale but were rather performed only for hospitalized individuals with COVID-19 at that time. The magnitude of connections to the web-based app was high in March 2020 because it occurred during the outset of media campaigns of the web-based app. No media campaigns occurred before the second wave of the pandemic in October 2020, which explains why the magnitude of the connections was lower than that during the first wave of the pandemic.

The second peak of daily reports of anosmia occurred in July 2020 without a subsequent increase in the number of hospitalizations. This may be explained by the young age of the infected users (median age 32 years), who were at a low risk of severe disease, and the summer season during which transmission to older individuals was limited by the elevated outdoor temperature and reduced indoor transmission. However, the reduction in the number of hospitalizations after the first wave of the pandemic decreased simultaneously, suggesting the initiation of a new outbreak in July 2020.

During the second wave of the pandemic in October 2020, the reduction in the positive outcomes on daily RT–PCR tests occurred only 2 days before the peaks in daily hospitalizations and ICU admission, suggesting a decreased potential of RT–PCR data to predict the precedence of the reduction in the number of hospitalizations when compared with the number of daily reports of anosmia. This issue was probably also associated with the delays in getting tested and in obtaining the results. Moreover, the analytic sensitivity of RT–PCR testing is high, and the long duration of the RNA-positive tail suggests that most infected individuals are being identified after the infectious period has passed. This may overestimate incidence of the disease and explain the lack of an association of daily testing with the peak in hospitalizations and ICU admission [7]. This overestimation is also associated with the high proportion of asymptomatic individuals identified through contact tracing investigations and self-testing of suspected individuals. The risk of saturation of biological laboratories and the lack of reactions on RT–PCR testing may also lead to the discordance of data from individuals testing positive from the actual dynamic of the outbreak, leading to the decreased potential of RT–PCR test data in predicting the hospitalization rate.

The potential of the web-based app as a predictor of the hospitalization rate is based on the high specificity of anosmia as a diagnostic feature of COVID-19, occurring few days before symptom exacerbation among hospitalized patients with COVID-19 [6].

We recorded a high peak of daily reports of anosmia on September 16, 2020, which was 49 days before the maximum peak of daily hospitalizations in November 2020, with a low median age of users (9.1% [n=190/2088] of whom were aged ≥45 years); this was followed by a second peak of hospitalizations among older individuals on October 26, 2020 (24.1% [n=402/1668] of whom were aged >45 years). The anticipation of the peak in hospitalizations in these 49 days is probably specific to the summer period and subsequently to a relatively large first wave of infections among young adults—which occurred in mid-July 2020 without a significant increase in the hospitalization rate—and at a higher scale toward the end of summer vacations and during the back-to-school period after which many clusters of infection were observed at schools and universities. As young, internet-savvy users developed anosmia, they extensively used the app, but only few of them had severe COVID-19 and few required subsequent hospitalization. The September 2020 peak in daily reports of anosmia was probably followed by progressive disease transmission to older patients in October 2020, with a subsequent domino effect during low-temperature periods and increased indoor transmission. This may have triggered a second wave of infections among older users (who are at an increased risk of severe COVID-19) in October 2020, followed by a large wave of hospitalizations in November 2020. Since this app is used less extensively by older patients (in whom anosmia is less frequent), the magnitude of the daily reports of anosmia was lower in October 2020 than in September 2020, but the hospitalization rate increased. Thus, this app does not predict the magnitude of hospitalization after a peak in the reports of anosmia, unless focusing on older individuals.

During the first and the second wave of the pandemic in France, a reduction in the daily reports of anosmia preceded the reduction in the number of daily hospitalizations and ICU admissions by 10 and 9 days, respectively, in March and October 2020 but not in July or September 2020. This was not observed in July 2020 after a low hospitalization rate was reported among older individuals. However, daily hospitalizations stopped increasing in September 2020 a few days after the reduction in reports of anosmia in September 2020.

Although this tool does not accurately anticipate an increase in the magnitude of hospitalization, it seems to accurately predict the reduction in the hospitalization rate. The anticipation of the reduction in emergency department visits individuals reporting anosmia (by 7 days), hospitalizations (by 9 days), and ICU admissions (by 9 days) is thus crucial for anticipating the surge duration during the pandemic and in managing the requirements of beds in the ICU and dedicated COVID-19 wards.

We recorded more than 13,000,000 responses between March 17 and November 18, 2020, and 575,214 daily reports of anosmia from among an unknown number of users. As user data were anonymized, duplicate responses may have been obtained and were not assessable. However, the anonymous nature of the data collected from the app and the lack of tracking favored its extensive utilization in the French population, which was not observed with tracking applications.

As revealed from RT–PCR data, the infection rates decreased 15 days after large-scale curfew and lockdown measures were enforced, whereas daily reports of anosmia began decreasing 4 days later. This observation, along with spatiotemporal data obtained from reports of anosmia, can help the authorities nationwide to harness the value of emergency department visits, hospitalizations, and ICU admissions and to obtain early estimates of the outcomes of lockdown measures. Studies have assessed the generalizability of this tool in many countries in Europe (eg, UK and Germany) and in the United States [8-11].

## Abbreviations

ICU: intensive care unit
